# Effect of synthetic bone replacement material of different size on shear stress resistance within impacted native and thermodisinfected cancellous bone: an in vitro femoral impaction bone grafting model

**DOI:** 10.1007/s10561-021-09924-w

**Published:** 2021-04-24

**Authors:** C. Fölsch, P. Sahm, C. A. Fonseca Ulloa, G. A. Krombach, M. Kampschulte, M. Rickert, A. Pruss, A. Jahnke

**Affiliations:** 1 Department of Orthopaedic Surgery, Justus-Liebig-University Medical School, Klinikstrasse 33, 35392 Gießen, Germany; 2 Laboratory of Biomechanics, Department of Orthopaedic Surgery, Justus-Liebig-University Medical School, Klinikstrasse 29, 35392 Giessen, Germany; 3 Department of Diagnostic and Interventional Radiology, Laboratory for Experimental Radiology, Justus-Liebig-University Medical School, Klinikstrasse 33, 35392 Giessen, Germany; 4 Institute of Transfusion Medicine, University Tissue Bank, Charité University Medical School, Charitéplatz 1, 10117 Berlin, Germany

**Keywords:** Bone bank, Thermodisinfection, Infection joint replacement, Allogeneic bone, Femoral impaction bone grafting, Local antibiotic therapy, Bone reconstruction

## Abstract

Antibiotic carrier particles of variable size might influence mechanic properties within impacted thermodisinfected and native cancellous bone different. Herafill®G containing calciumsulfate and calciumcarbonate provides high local concentrations of gentamicin being important for revision surgery in infected joint replacements. Native and thermodisinfected cancellous bone derived from 6 to 7 months old piglets was used for in vitro impaction bone grafting and supplemented each with Herafill®G granules of two different sizes. Micromovement of implants related to shear force was measured in 29 specimens distributed in 6 groups. Thermodisinfected cancellous bone revealed a significant higher shear force resistance than native bone with a mean difference of 423.8 mdeg/Nm (*p* < 0.001) ranging within 95% confidence interval from 181.5 to 666.0 mdeg/Nm. Adding small granules to thermodisinfected bone did not reduce shear force resistance significantly since adding large granules to native bone improved it by 344.0 mdeg/Nm (*p* < 0.003). Shear force resistance was found higher at the distal region of the implant compared to a proximal point of measurement throughout all specimens. Less impaction impulses were necessary for thermodisinfected bone. Thermodisinfected cancellous bone might achieve a higher degree of impaction compared with native bone resulting in increased resistance against shear force since impaction was found increased distally. Supplementation of thermodisinfected bone with small granules of Herafill®G might be considered for application of local antibiotics. Large granules appeared more beneficial for supplementation of native bone. Heterogeneity of bone graft and technical aspects of the impaction procedure have to be considered regarding the reproducibility of femoral impaction bone grafting.

## Introduction

Femoral impaction bone grafting for restoration of bone stock in revision surgery of hip joint replacement appears beneficial for good long term results (Halliday et al. 2003; Howie et al. 2010; ten Have et al. 2012; Wilson et al. 2016) and bone transplantation seems useful for reconstruction of bone defects in endoprosthetic revisions at different joints (Rudert et al. 2015; Windhager et al. 2017). Allogeneic bone is necessary to match the demand for bone graft since thermodisinfected and other processed cancellous bone derived from human femoral heads might be used alternatively to unprocessed native bone (Cornu et al. 2003; Fölsch et al. 2018; Pruss et al. 2003). The increasing number of joint replacement revision surgery for infectious disease is challenging (Frommelt 2018; Li et al. 2013). Local application of antibiotics within impacted bone graft appears useful in case of prevention or treatment of infection (Coraca-Huber et al. 2016; Fölsch et al. 2015, 2016a; Lewis et al. 2012). Suitable carrier substances provide high local antibiotic concentrations (Frommelt 2018) and should improve mechanic properties of the impacted bone graft.

Differences of mechanic properties of impacted bone grafts appear related to heterogeneity of native as well as processed bone grafts and the operative technique of impaction bone grafting (Ahmed et al. 2018; Arts et al. 2007; Fosse et al. 2006a, 2006b). Processing of bone reduces fat and fluid of bone transplants and correlated with increased bone density (Fosse et al. 2006c; McKenna et al. 2013; Oakley and Kuiper 2006; Putzer et al. 2014b) since adding hydroxyapatite granules to allogeneic bone improved bone mineral density and bone volume fraction (Fujishiro et al. 2005; Munro et al. 2007; Phipps et al. 2005; Yano et al. 2000). Increased stiffness and compactness was found for processed compared with native cancellous bone (Cornu et al. 2003, 2004, 2009; Fosse et al. 2006a; Nguyen et al. 2013; Oakley and Kuiper 2006). The reduction of height of processed cancellous bone correlated with improvement of mechanic properties (Fosse et al. 2006a; Giesen et al. 1999) including an increase of rotational stiffness (Ohashi et al. 2009; Oakley and Kuiper 2006). Different impaction behavior and biomechanical properties of irradiated compared with native cancellous bone regarding the size of the particles were reported (Cornu et al. 2009) since good clinical results were shown for femoral impaction bone grafting using irradiated allogeneic bone (Howie et al. 2010). High mechanical load capacity of impacted bone below the femoral implant is crucial to prevent subsidence (Gie et al. 1993a; Goldman and Sierra 2017; Heyligers et al. 2014).

Supplementation of thermodisinfected and native bone with carrier particles for antibiotics of different size might influence impaction behavior of cancellous bone (Cornu et al. 2003, 2004, 2009; Fölsch et al. 2018) affecting shear force resistance which is a relevant parameter for mechanical properties (Dunlop et al. 2003). The setup of the impaction bone grafting model was related to previous studies (Fölsch et al. 2018; Putzer et al. 2014a) since morphology and mechanic behavior of impacted bone is resembled by porcine cancellous bone (Fölsch et al. 2016b). Native and thermodisinfected bone specimens each composed of a mixture of particles with different configuration which had revealed a comparable distribution were chosen for the study (Cornu et al. 2009; Fölsch et al. 2018). Micromovement of the stem within the bone graft was measured at a proximal and a distal point according to expected differences of impaction (Fölsch et al. 2018; Omoto et al. 2008). Deviating impaction behavior of thermodisinfected cancellous bone compared with native cancellous bone resulting in different shear force resistance might be expected (Cornu et al. 2003, 2004, 2009) since a marginal reduction of mechanic properties due to thermodisinfection has to be considered (Fölsch et al. 2016b; Pruss et al. 2003). The biomechanic preferrable composition of impacted native and thermodisinfected cancellous bone supplemented with antibiotic carrier particles (Herafill®G) of different size should be determined. The assumed different degree of impaction of thermodisinfected and native cancellous bone and the resulting influence on the interaction with antibiotic carrier substances (Herafill®G) of variable size should be examined.

## Materials and methods

The volume of the bone chips had been calculated considering the average diameter of a porcine femoral head (30 mm) and according to the inner diameter (25 mm) and the length of the cavity (180 mm) of transplantation. Cancellous bone was harvested from 230 femoral heads of 6–7 months old female and male piglets with a weight of 90 kg within 12 h after they had been slaughtered (Manz, Hüttenberg, Germany). The femoral heads were removed at the neck of femur using an oscillating saw (Multitalent FMT 250 SL, Fein, Schwäbisch Gmünd-Bargau, Germany). Remaining soft tissue was removed with a scalpel followed by immediate storage at − 20 °C. From the total number of 226 femoral heads 113 were randomized thermodisinfected and the other half remained native. Thermodisinfection was performed with the Marburg bone bank procedure (Lobator sd-2, Telos GmbH, Marburg, Germany). Each procedure was done with 3 thawed femoral heads being placed in isotonic normal saline solution (0.9% Sodium-Chloride, Sigma-Aldrich, St. Louis, Missouri, United States). The heat application took 94 min and applied at least 82.5 °C for 15 min within the center of the femoral head.

**Fig. 1:**
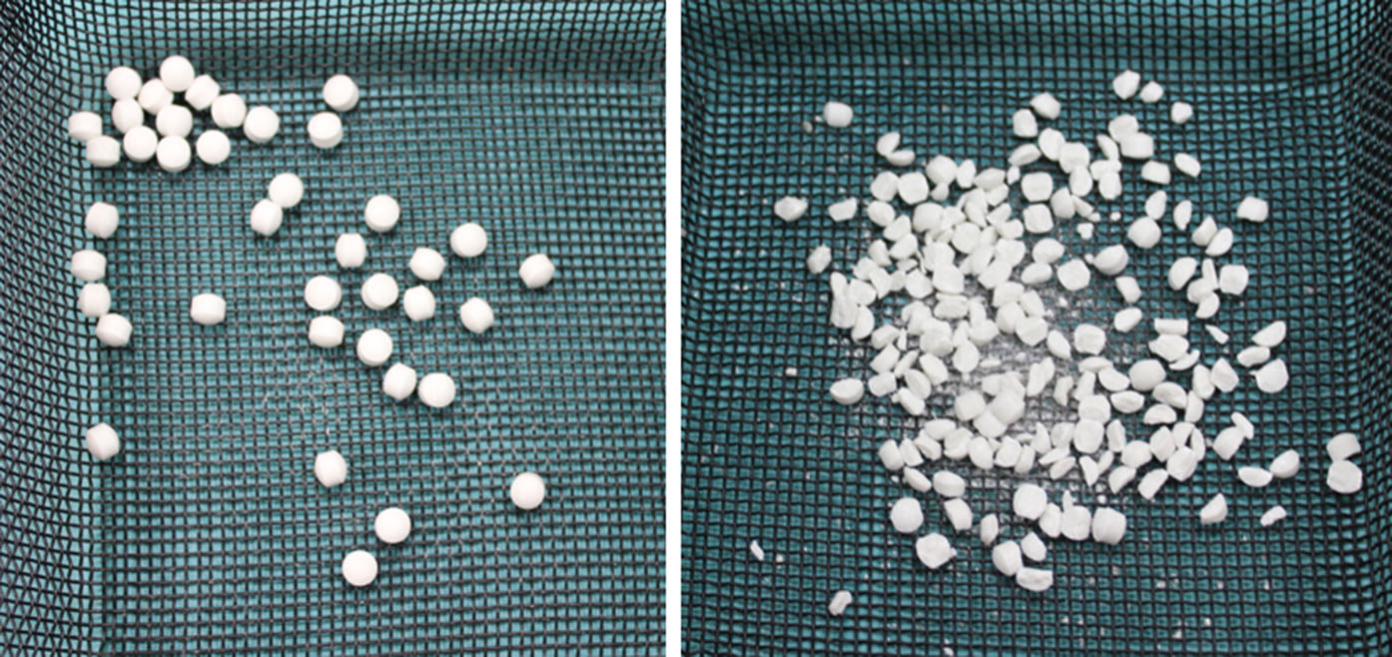
Complete antibiotic pellets (large granules, left) and smashed pellets (small granules, right) to supplement bone graft

**Fig. 2:**
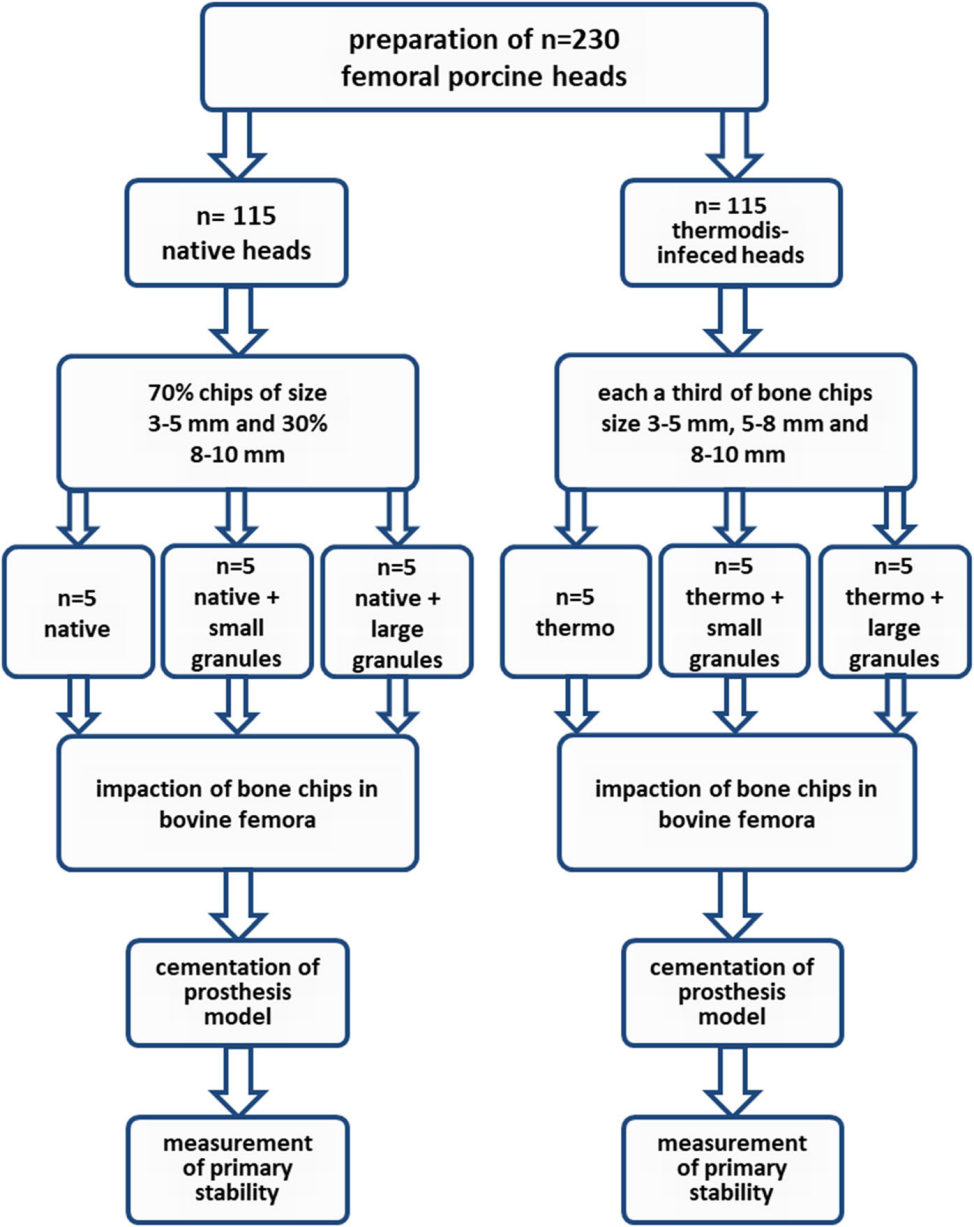
Outline of the experiment structure showing the composition of the different test samples of native and thermodisinfected bone combined with small (2 mm to 5 mm) and large (5 mm to 6 mm) granules of Herafill®G

One group of native and thermodisinfected cancellous bone specimens and a combination of each with two different geometric forms of synthetic bone material either entirely measuring 5 to 6 mm or fragmented with variable size of particles between 2 mm and less than 5 mm were examined (Herafill® beads G, Heraeus Medical GmbH, Wehrheim, Germany) (Figs. 1, 2) which consists of calciumsulfate-dihydrate, calciumcarbonate, hydrogenated triglyceride und gentamicinsulfate. The smaller pellets were generated smashing within a protection bag consisting of polyamide-polyethylen and fragments smaller than 2 mm were removed with a 2 mm sieve.

According to statistical analysis (Power-Analysis G*Power Vers. 3.1.9.2., HHU Düsseldorf, Germany) based on data of a comparable study (Fölsch et al. 2016b) with α (5%), β (20%) and mistake (Power 80%) as well as relative movements of 15.2 and 15.8 (SD = 0.2) mdeg/Nm the necessary number of specimens within each group was calculated n = 4 and n = 6 was chosen. Therefore 36 models were created.

Following rinsing the femoral heads in normal saline solution (0.9%) at 21 °C (± 1 °C) for three hours (Fölsch et al. 2018) bone chips with defined size of 3–5 mm and 5–8 mm as well as 8–10 mm were manufactured (Noviomagus Bone Mill, Spierings Orthopaedics Nijmegen, Netherlands). According to a previous study (Fölsch et al. 2018) the composition with the resulting best distribution of native and thermodisinfected cancellous bone chips following the impaction was used for the examination. The native cancellous bone graft consisted of 70% bone chips of size 3–5 mm and 30% 8–10 mm since the thermodisinfected bone graft contained each a third of bone chips size 3–5 mm and 5–8 mm as well as 8–10 mm (Fig. 2). The bone graft included either 40 smashed or 40 entire Herafill®G particles within each composite group (Fig. 1).

**Fig. 3:**
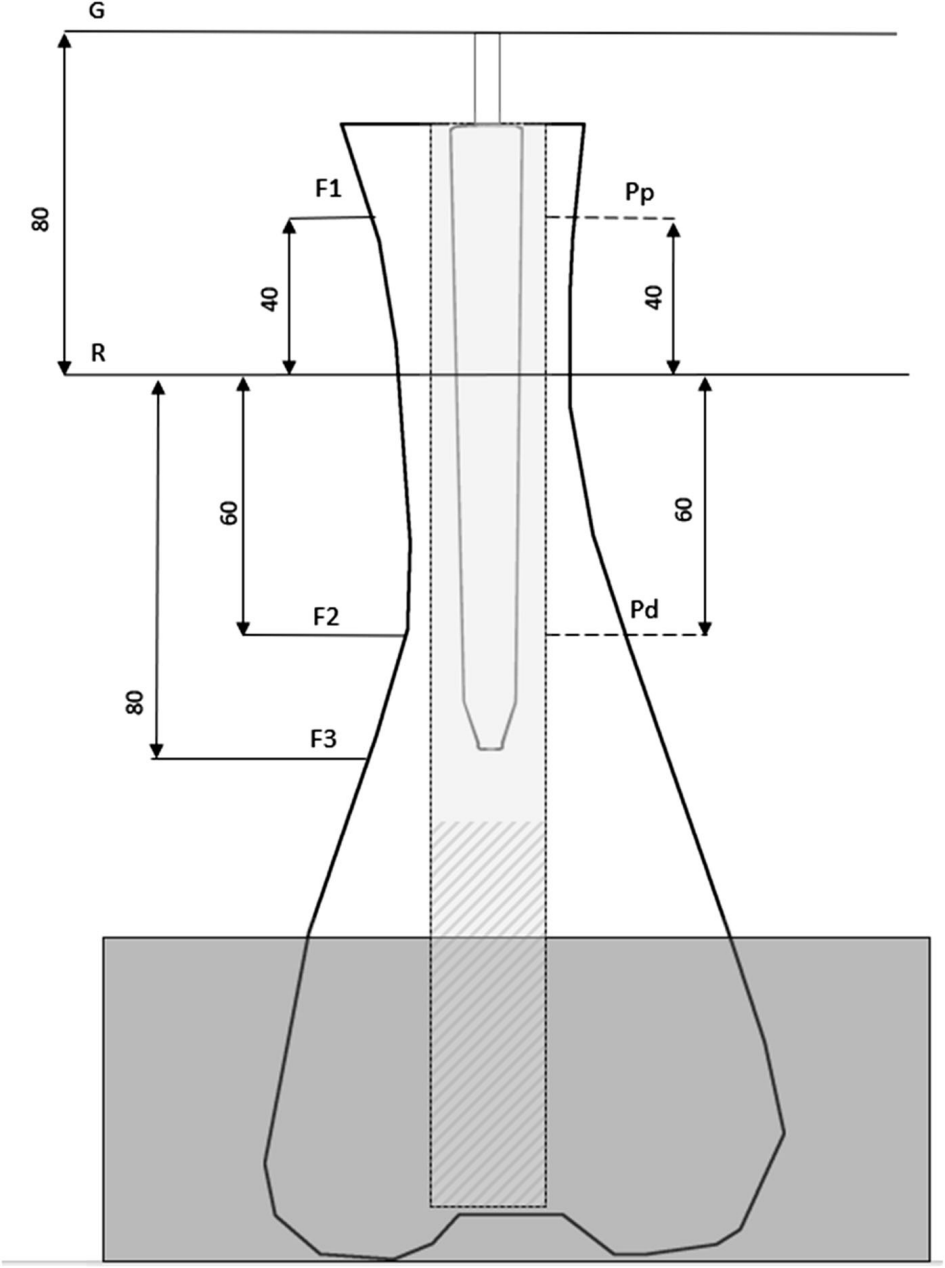
Model of experimental setup and definition of relevant markings: height overall (G), height of reference level (R), measurement levels of bone micromovement (F1, F2, F3) and prosthesis micromovement (Pp, Pd)

**Fig. 4:**
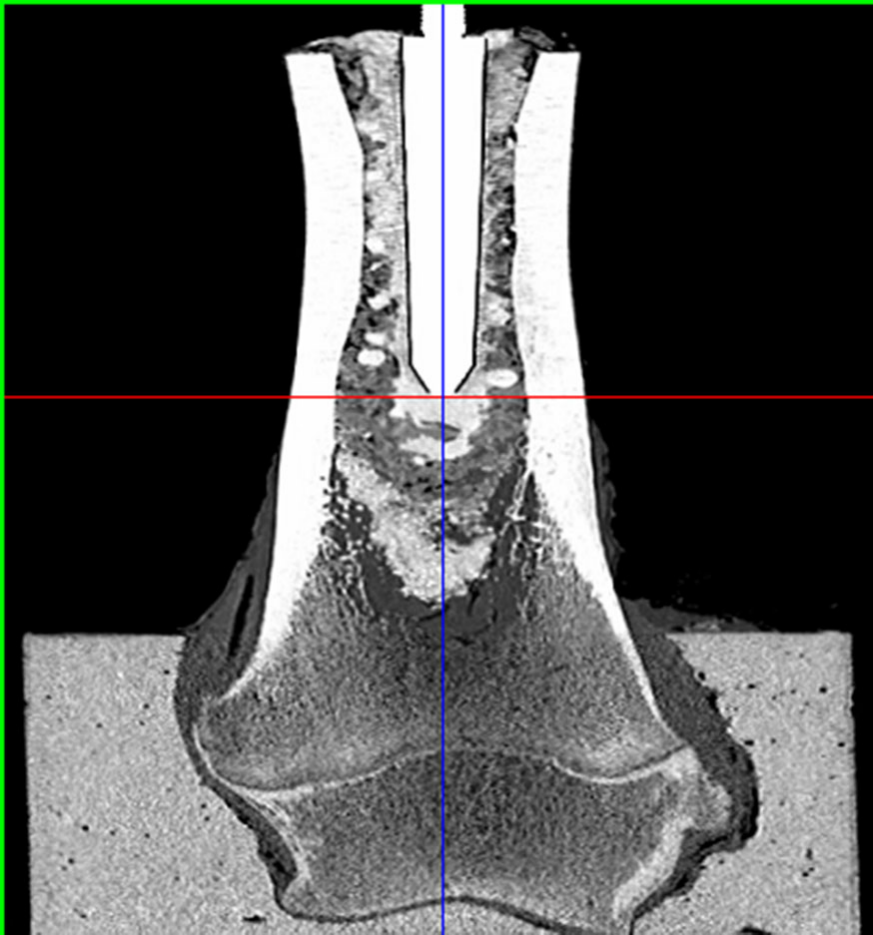
Computertomographic study of a native specimen mixed with large granules

Bovine femora were harvested from 38 eighteen months old cattles with a weight between 550 and 650 kg (LahnFleisch GmbH & Co. KG, Wetzlar, Germany). The femora were stored at -20 °C and the diameter at the junction between diaphysis and metaphysis was 6 cm with a length of 45 cm. After 16 h of thawing remaining soft tissue was removed and a horizontal osteotomy was performed submetaphyseal using the oscillating saw. The length of the bone model ranged from 24.8 to 38 cm and a reference point was taken 80 mm below the top (Figs. 3, 4). Bone marrow and blood vessels were removed without damaging the compact bone. To achieve a standardized level of 17 cm within the marrow the bottom was filled with sand of grain size up to 2 mm. The femur was orientated horizontally and vertically using a self-leveling laser (Quigo, Robert Bosch Power Tools GmbH, Leinfelden-Echterdingen, Germany) and fixed with plaster in an aluminium socket.

The different mixture with native and thermodisinfected cancellous bone was thawed in 21 °C ± 1 °C normal saline solution for 10 min and then the bone chips were put into a towel (Telasorb®, Hartmann AG, Heidenheim, Germany) for rinsing procedure to reduce fat and water content. Bone particles which were smaller than 2 mm were removed with a sieve.

**Fig. 5:**
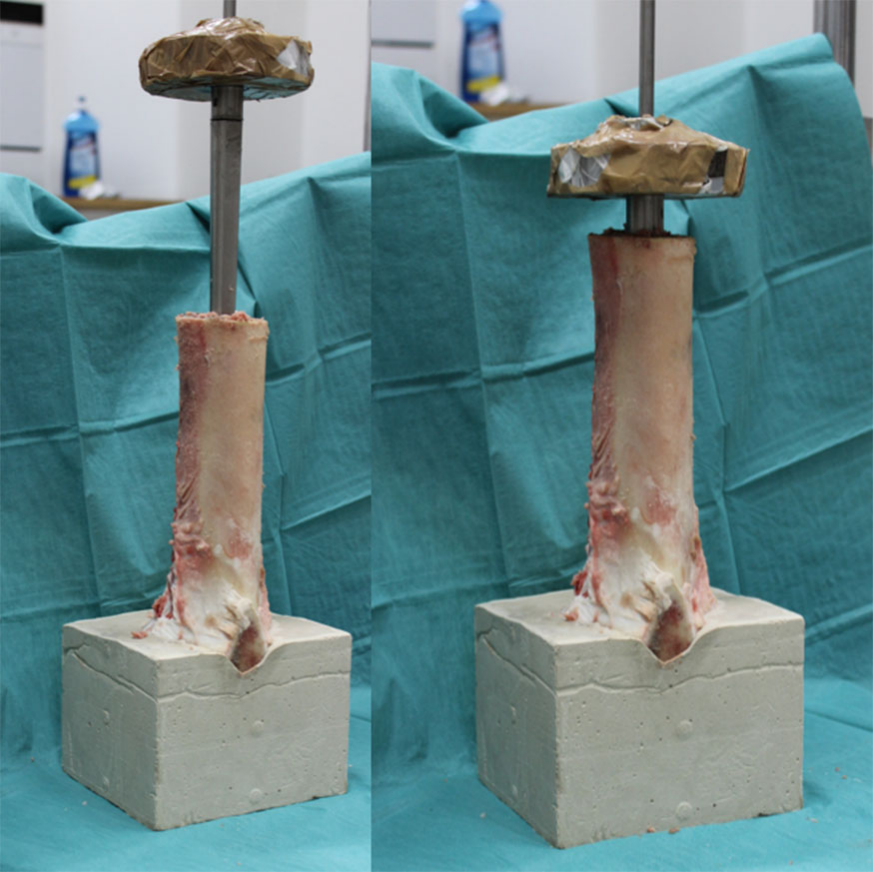
Weight and setup for impaction procedure

The filling of the cavity with native bone chips (groups 1–3) was performed in three steps since the thermodisinfected bone was filled entirely within one procedure (groups 4–6) (Fig. 2). Each 40 complete and smashed pellets (Herafill®G) were added to groups 2, 3, 5 and 6. The impaction was done with a weight of 803 g from a height of 218 mm providing a standardized impaction procedure (Fig. 5). The impactor itself was sprayed with silicon to ensure removal after impaction. The number of impactions was recorded until the impactor reached the edge of the femur.

Cement (Palacos®, Heraeus Medical GmbH, Wehrheim, Germany) was mixed under vacuum (Palamix®, Heraeus) of 200 mbar within 30 s and was then retrogradely filled into the preformed cavities of cancellous bone. Before implantation the shaft was defatted with 1-Propanolol and then inserted to similar depth of the impactor. Fluid was removed from the surface of the native bone as well as excess cement. After hardening of the cement during 15 min the specimens were stored again at − 20 °C.

**Fig. 6:**
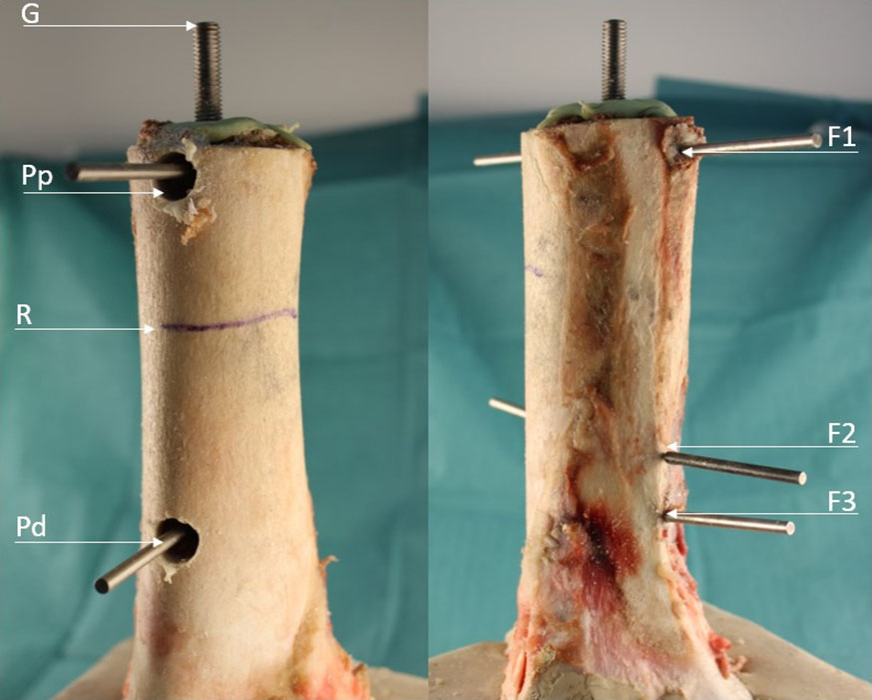
Points of measurement of micromovement on bone specimens and defined points corresponding to Fig. 2

The models were measured after 16 h of thawing and two points at the ventral prostheses P_P_ and P_D_ and three points at the dorsal femur F_1_–F_3_ were defined correlated to a reference point R 80 mm below the top level (Fig. 6). The ventral cortical bone was drilled with a 12.5 mm drill (SBEV 1000–2 Metabowerke GmbH, Nürtingen, Germany) followed by drilling the cement mantle with a 1.9 mm drill (LWB/E, PROXXON S.A., Wecker, Luxemburg) to allow fixation of measurement pins with cyanoacrylat. The corresponding dorsal femur points were drilled with a 1.9 mm drill.

**Fig. 7:**
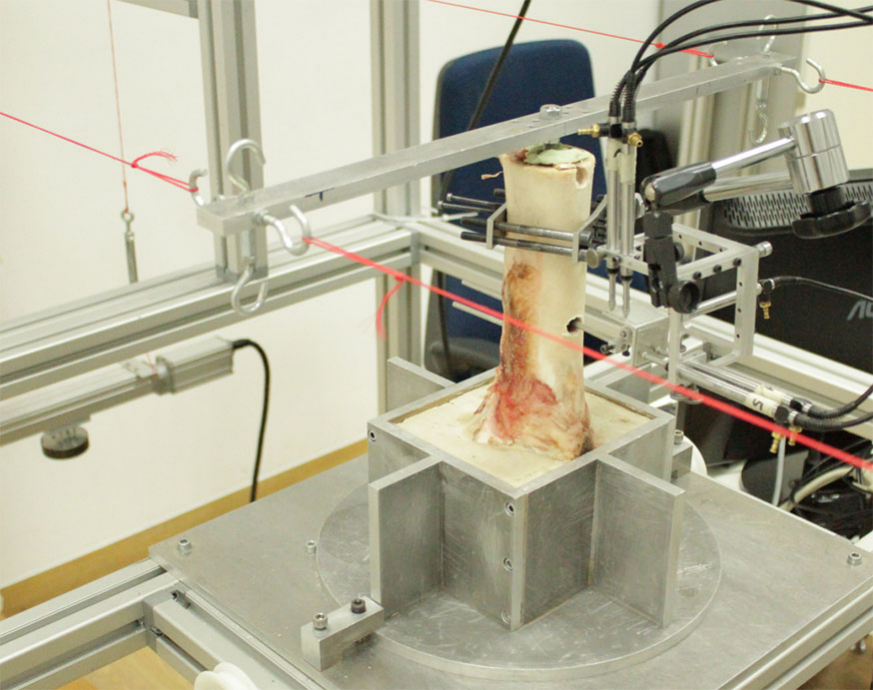
Experimental setup and measurement device for relative micromovement

Relative micromovements between bone and prosthesis-cement compound were recorded (Jahnke et al. 2020) and cycling torsional torques in a non-destructive range of ± 0.37 Nm (Fölsch et al. 2016b) were applied to examine the normalized rotational stability. A measuring pin was attached to the individual measuring points to which a measuring cube made of aluminum was fixed vertically. This measuring cube was located within an outer measuring frame in which six inductive displacement sensors were located in a 3–2-1 arrangement with a resolution of 0.1 µm (P2010, Mahr GmbH, Göttingen, Germany) (Fig. 7). With these displacement sensors the relative micromovements of the prosthesis-cement composite and the bone were then consecutively recorded for each individual measuring point. The relative micromovement (mdeg/Nm) at the measurement points rm1 and rm2 resulted from the difference between these movements.

Statistical analysis was done with SPSS Statistics® (Version 26.0, IBM, Armonk, New York, USA). Average values of relative micromovements (mdeg/Nm) and standard deviation (SD) between prosthesis-cement compound and cortical bone were calculated taking into account the measurement locations rm_1_ (P_p_ and F_1_) and rm_2_ (P_d_ and F_2_) and the composition of cancellous bone. Standard error (SE) was calculated for differences and 95% confidence interval. Analysis of variance was done with a generalized linear model. The LSD Post-Hoc-Test as well as the Bonferroni adjustment was applied.

## Results

**Fig. 8:**
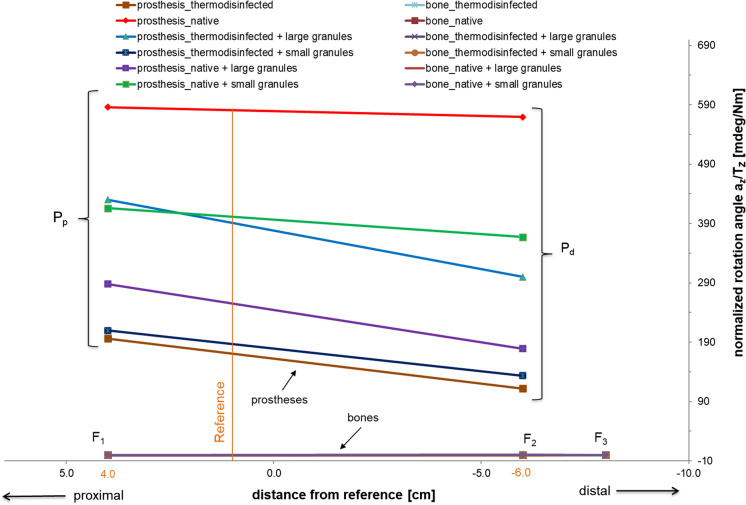
Motion graph of all experimental groups. The measuring level is plotted on the abscissa (proximal = positive direction, distal = negative direction) and the normalized rotation angle α_Z_/T_Z_ (mdeg/Nm) is plotted on the ordinate. The upper lines schematically represent the prostheses and the lower lines the femora. Due to no perceived movement in the respective bone graft the bone characteristics overlap

Measurements were performed on 29 bone specimens (Tables 1, 2) which are shown in the movement graph of all prosthesis-cement composites in relation to the corresponding bone (Fig. 8) since 7 specimens appeared prone to failure during implant testing. None of the composites revealed relative micromovements in the corresponding bone showing no force transmission into the impacted bone graft. Therefore the movement curves of the bones are displayed superimposed (Fig. 8). For the impaction of the bone material a variable number of impulses were applied showing a tendency to less number of impactions for preparation of the thermodisinfected specimens (Table 1). The impacted thermodisinfected cancellous bone showed a significant higher shear force resistance compared with native bone with a mean difference of 423.8 mdeg/Nm ± 120.3 mdeg/Nm (SE) (*p* < 0.001) (Table 2) since the 95% confidence interval of the difference was ranging from 181.5 to 666.0 mdeg/Nm.

**Table 1:** Number of impactions for impaction bone grafting of different groups of specimens

Group	Material	Number of impactions					
1	Native cancellous bone	64	71	73	25	32	44
2	Native cancellous bone and small granules	32	66	36	42	38	32
3	Native cancellous bone and large granules	46	27	20	45	70	70
4	Thermodisinfected cancellous bone	32	50	15	21	31	30
5	Thermodisinfected cancellous bone and small granules	18	32	27	40	37	51
6	Thermodisinfected cancellous bone and large granules	66	44	27	28	32	29

**Table 2:** Measurements of micromovement (mdeg/Nm) including standard deviations (SD) at two measurement levels (rm1, rm2) and average values with the number of bone specimens (N) of all groups (Table 1)

Group	Measurement	Average	SD	N
1	Proximal (rm1)	586.0	465.6	3
	Distal (rm2)	569.4	398.8	3
	both points	577.7	387.9	6
2	Proximal (rm1)	415.8	185.6	6
	Distal (rm2)	367.0	199.9	6
	both points	391.4	185.7	12
3	Proximal (rm1)	288.2	226.1	6
	Distal (rm2)	179.1	143.5	6
	both points	233.7	189.3	12
4	Proximal (rm1)	196.0	66.8	4
	Distal (rm2)	111.9	137.9	4
	both points	153.9	109.9	8
5	Proximal (rm1)	209.4	103.1	5
	Distal (rm2)	133.4	139.7	5
	both points	171.4	122.5	10
6	Proximal (rm1)	429.9	241.0	5
	Distal (rm2)	300.0	307.7	5
	both points	365.0	269.4	10

Adding small granules to thermodisinfected bone did not increase the micromovement significantly with 17.5 mdeg/Nm ± 105.7 mdeg/Nm (SE). A significant difference of 406.3 mdeg/Nm ± 115.1 mdeg/Nm (SE) was measured between native bone and the mixture of thermodisinfected bone with small granules (*p* < 0.001) since the 95% confidence interval of the difference was ranging from 174.6 to 637.9 mdeg/Nm. Adding small granules to native bone decreased the micromovement not significantly with 186.3 ± 111.4 mdeg/Nm (SE) (Table 2). The difference between native bone mixed with small granules and thermodisinfected bone appeared significant with 237.4 ± 101.7 mdeg/Nm (SE) (*p* = 0.024) (Table 2) since the 95% confidence interval was ranging from 32.7 to 442.2 mdeg/Nm. Thermodisinfected and native bone each mixed with small granules revealed a significant difference of 219.9 ± 95.4 mdeg/Nm (SE) (*p* = 0.026) (Table 2) since the micromovement measured for native bone supplemented with small granules remained higher (Table 2, Fig. 8).

A difference of 344.0 ± 111.4 mdeg/Nm (SE) was found between native bone and its mixture with large granules which reduced micromovement significantly (*p* < 0.003) (Table 2). Addition of large granules to thermodisinfected bone increased micromovement by 211.0 ± 105.7 mdeg/Nm (SE) without significant difference (*p* = 0.052). No significant difference of 131.3 ± 95.4 mdeg/Nm (SE) was observed between native and thermodisinfected bone each mixed with large granules since the micromovement was found higher for the mixture with thermodisinfected bone (Table 2). The 95% confidence interval of the values ranged between 223.1 and 506.8 mdeg/Nm for thermodisinfected and from 104.2 to 363.1 mdeg/Nm for native bone each mixed with large granules.

The proximal measurement points showed higher micromovements compared with the distal areas throughout all groups (Table 2) (Fig. 8) and the 95% confidence interval ranged from 268.4 to 440.0 mdeg/Nm proximal and from 191.0 to 362.6 mdeg/Nm distal since the mean difference between both points of measurement was 77.4 ± 60.3 mdeg/Nm (SE). No significant differences were found between the proximal and distal point of measurement in each group. A smaller difference between the proximal and distal point of measurement was found in the native group compared with thermodisinfected specimens since the standard deviation appeared smaller for thermodisinfected bone at the proximal and distal point compared with the native specimens (Table 2).

Mixture with small and large granules reduced micromovement in the native cancellous bone group at both points of measurement in all groups since the movement was increased for thermodisinfected bone (Table 2). Thermodisinfected specimens showed a small increase of micromovement of 13.5 mdeg/Nm proximal and 21.5 mdeg/Nm distal after addition of small granules since the movement increased further with mixture of large granules (Table 2). At the proximal point an increase of micromovement to 234.0 mdeg/Nm was measured for the mixture of large granules with thermodisinfected bone and 188.1 mdeg/Nm were found at the distal point. A small difference was calculated for native bone and its mixture with small granules according to values of 170.2 mdeg/Nm proximal and 202.5 mdeg/Nm micromovement distal which appeared reduced compared with native bone alone (Table 2). Adding large granules to native bone reduced micromovement by 297.8 mdeg/Nm proximal and 390.3 mdeg/Nm distal (Table 2). Singular computertomographic studies showed a comparable regular distribution of the impacted bone grafts of all groups (Fig. 4).

## Discussion

Heterogeneity of the cancellous bone and differences of the impaction technique have to be considered regarding mechanic properties of impacted bone graft (Albert et al. 2008; Fosse et al. 2006b, 2006c; Frei et al. 2004; Phillips et al. 2006b) since the number of impaction procedures seemed to be related to the quality of bone (Ahmed et al. 2018; Bavadekar et al. 2001; Fölsch et al. 2018; Oakley and Kuiper 2006). The necessary number of impaction impulses for thermodisinfected bone appeared less compared with native bone and fluid needed to be removed from the top of the native specimens following impaction (Table 1). Liquids lubricating from the native bone specimens during impaction should favor particle movement resulting in increased density of the material which appears enhanced by reduced viscosity related to low fat content (Fosse et al. 2006b, 2006c). The bone volume applied for impaction bone grafting was found increased for thermodisinfected bone indicating more compactness compared with native cancellous bone (Fölsch et al. 2018). The degree of impaction correlated with mechanic stability (Albert et al 2008; Fosse et al. 2004, 2006b, 2006c) and inversely with porosity of bone (Frei et al. 2004). Relevant differences of mechanic properties of impacted bone were reported depending on different impaction procedures (Bavadekar et al. 2001; Fosse et al. 2004; Phillips et al. 2006a, 2006b) since cortical bone grafts reduced subsidence of femoral stems (Kligman et al. 2003; Ohashi et al. 2009; Omoto et al. 2008; Putzer et al. 2014b). According to processing bone grafts with thermodisinfection an impairment of mechanic stability and alteration of the impaction behavior could be expected (Cornu et al. 2003; Fölsch et al. 2016b; Fosse et al. 2006b, 2006c) and less numbers of impaction impulses indicate a different impaction behavior of native and thermodisinfected cancellous bone (Fölsch et al. 2018). A negative influence of thermodisinfection on shear force resistance of impacted cancellous bone was not shown since significant less micromovement of thermodisinfected compared with native bone was found (Table 2, Fig. 8). Reduced movement within the impacted bone graft distal (Table 2) might be related to different distribution of energy during femoral impaction grafting (Fosse et al. 2006a; Frei et al. 2005a, 2005b). For particles being more prone to viscoplastic behavior than native bone like processed thermodisinfected bone the impaction force seems more important for the impaction than the particle size (Albert et al. 2008). Native and thermodisinfected bone particles of different size achieved comparable distribution of bone graft assuming different mechanical properties (Cornu et al. 2009; Fölsch et al. 2018). The influence of multiple factors on the reproducibility of impaction bone grafting has to be considered regarding the measured biomechanic difference between native and thermodisinfected cancellous bone. Since less impactions appeared necessary for thermodisinfected bone comparable to other studies on processed bone the wide range of the measured values reflects the heterogeneity of the bone grafts (Table 2) (Cornu et al. 2003, 2004, 2009). The higher shear force resistance for impacted thermodisinfected cancellous bone indicates that the alteration of the mechanic properties due to thermodisinfection might be balanced during impaction. The different impaction behavior is also reflected by the small increase of shear force resistance from the proximal to the distal point of measurement for the native bone compared with the large difference in thermodisinfected bone since the degree of impaction appeared increased distal in both groups (Table 2) (Fölsch et al. 2018).

The size of the bone particles showed an influence on the impaction and distribution of native (Albert et al. 2008; Cornu et al. 2009; Fosse et al. 2006a; Kligman et al. 2003; Putzer et al. 2014a) and thermodisinfected cancellous bone (Fölsch et al. 2018) since movement of particles seems to be relevant to obtain a good stability during impaction (Putzer et al. 2011). Smaller bone particles were recommended in femoral impaction bone grafting distally since the ideal particle size remains in discussion (Goldman and Sierra 2017; Heyligers et al. 2014; Scanelli and Brown 2013). Particles need to be interlocked to improve stiffness (Fosse et al. 2006c; Putzer et al. 2014a). The addition of small granules to thermodisinfected bone did not reveal a significant difference of shear force resistance compared with pure processed bone indicating no relevant disturbance of the impaction since that mixture also remained significantly different from native bone (*p* = 0.001) (Table 2). A significant difference between native and thermodisinfected bone each mixed with small particles was found (*p* = 0.026) (Table 2) since a wide range of values according to the 95% confidence intervall has to be considered. The shear force resistance of large granules mixed with native bone appeared higher compared with its addition to thermodisinfected bone (Table 2, Fig. 8). The increase of micromovement of thermodisinfected bone and its mixture with larger particles did not appear significant (*p* = 0.052) which might be related to the number of specimens since the addition of large granules to native bone reduced micromovement significantly (*p* = 0.003). An impairment of the impaction of thermodisinfected cancellous bone due to adding large granules could be assumed since an improvement for native bone might be expected (Cornu et al. 2009). That different behavior of the native bone might be related to interaction with the surface of the supplemented particles and its deformation properties (Atencia and Beebe 2005; Cornu et al. 2009; Oakley and Kuiper 2006). The size of the particles was shown to be relevant for the stability of the impacted cancellous bone (Cornu et al. 2009; Fosse et al. 2006a, b; Putzer et al. 2014a) since variations in size and shape appeared beneficial (Cornu et al. 2003, 2009; Giesen et al. 1999). The mixture of small and large granules both leveled the difference of movement between native and thermodisinfected bone since the influence of large particles on impaction behavior appeared pronounced (Table 2, Fig. 8). A different effect on the impaction behavior of native and thermodisinfected cancellous bone should be considered since less change of mechanic properties of impacted bone due to smaller particles was shown according to clinical studies (Cornu et al. 2003; Gehrke et al. 2013). In the early phase of impaction fluid is part of transfer of load followed by viscoelastic and viscoplastic deformation of bone (Albert et al. 2008). This might be relevant regarding different content of water within impacted bone graft and the removal of fluid from native specimens (Fölsch et al. 2018). Increased adhesion might be related to the different influence of large particles on shear force resistance of impacted native bone (Fölsch et al. 2018; Oakley and Kuiper 2006).

Porosity of impacted native and processed cancellous bone decreased towards the tip of the stem since the performance of impaction was not found correlated with the degree of impaction (Fölsch et al. 2018; Fosse et al. 2004, 2006b, c; Frei et al. 2005a; Ohashi et al. 2009; Phillips et al. 2006b) (Table 2). Reduced micromovement distal might be related to the increased density of impacted native and processed cancellous bone (Cornu et al. 2009; Fölsch et al. 2018). Cement intrusion in the distal two thirds of impacted bone graft seemed mainly determined by graft permeability and appeared reduced distal in thermodisinfected compared with native cancellous bone (Fölsch et al. 2018; Frei et al. 2004, 2006) which might reflect reduced porosity of impacted thermodisinfected cancellous bone in the distal region (Fig. 8) (Frei et al. 2005b) since increased air in the proximal region of thermodisinfected cancellous bone was shown (Fölsch et al. 2018). This could be related to differences of micromovement at the proximal and distal point of measurement in the thermodisinfected bone specimens compared with native bone (Ohashi et al. 2009) (Table 2). Less micromovement at the distal point of measurement was a consistent behavior of impacted native and thermodisinfected cancellous bone also being supplemented with small and large granules (Table 2). The cement penetration into the bone allograft and the cement volume resulting in an increased surface were found higher for native than for thermodisinfected cancellous bone (Fölsch et al. 2018; Frei et al. 2005b). The influence of increased density on primary stability of impacted bone and the penetration of cement into the bone graft have to be considered. Penetration of cement was found impaired in the distal area of implanted stems since failure of specimens appeared within the impacted bone graft indicating a stable cement fixation (Coathup et al. 2008; Frei et al. 2005b) since significant cement penetration into the bone graft could interfere with bony integration (Migaud et al. 2008).

Quality and shape of bone grafts were shown to influence deformation behavior during impaction bone grafting (Fosse et al. 2006b, 2006c; Giesen et al. 1999) and in the proximal region cement distribution appears mainly related to the shape of particles (Fölsch et al. 2018; Frei et al. 2004, 2006; Ohashi et al. 2009). The impaction of processed irradiated bone grafts achieved more stiffness than native bone and structurally altered bone revealed a reduced elastic modulus (Cornu et al. 2003, 2009) since different motion of implants was found related to the dose of applied irradiation (Costi et al. 2013). Freeze dried bone provided more stability for cemented stems than fresh frozen bone graft (Cornu et al. 2003, 2009, 2011). The degree of impaction correlated inversely with porosity (Frei et al. 2004) and more deformation energy was absorbed by native bone (Cornu et al. 2003, 2009). The increased incorporation of air within the proximal region of impacted thermodisinfected cancellous bone and the larger difference of micromovement between the proximal and distal point of measurement compared with native bone might indicate local differences of the impaction (Fölsch et al. 2018; Ohashi et al. 2009) (Table 2). Similar to other processing procedures of bone grafts impaction bone grafting of thermodisinfected cancellous bone might achieve a comparable or increased shear force resistance compared with native bone (Bavadekar et al. 2001; Cornu et al. 2003, 2009; Costi et al. 2013). The small applied torque has to be considered since shear force resistance might vary between the bone composites depending on the applied force.

The heterogeneity of the bone graft and technical aspects of the impaction procedure affecting the reproducibility of impaction bone grafting have to be considered (Albert et al. 2008; Fölsch et al. 2018; Ohashi et al. 2009). The addition of large and small granules of variable size revealed a different influence on impaction behavior of native and thermodisinfected cancellous bone leveling the difference of shear force resistance between them (Fig. 8). Addition of small granules to native and thermodisinfected impacted bone caused no significant change of mechanic behavior. Adding large particles to native impacted bone improved shear force resistance significantly and reduced it relevant for thermodisinfected bone indicating a pronounced influence of large granules on impaction behavior. This might be related to different interaction between the particles (Fosse et al. 2006c). Small granules should be preferred as carrier for antibiotics within impacted thermodisinfected cancellous bone since supplementing native bone with large granules should be beneficial. Regarding the heterogeneity of the bone graft as well as the influence of multiple technical factors on the impaction procedure small granules might also be feasible for clinical application in combination with native bone.

A mechanically stable distal fixation is important for the primary stability of the femoral implant to avoid subsidence and allow reconstruction of the bone stock (Heyligers et al. 2014; Migaud et al. 2008). Femoral impaction bone grafting based on the technique described by the working groups from Exeter and Nijmegen (Gie et al. 1993a, 1993b) might be indicated in case of femoral defects Paprosky 3B and 4 in particular for younger patients (Goldman and Sierra 2017; Heyligers et al. 2014; Scanelli and Brown 2013; ten Have et al. 2012). Native cancellous bone chips of size 2 mm to 8 mm were recommended for distal impaction (Goldman and Sierra 2017; Heyligers et al. 2014) since larger particles seemed preferrable for proximal reconstruction (Scanelli and Brown 2013). Impaction of thermodisinfected bone should be beneficial for bone grafting distal and around the tip of the implant since native cancellous bone seemed preferrable in proximal regions (Fölsch et al. 2018; Frei et al. 2004, 2006; Ohashi et al. 2009). Impaction of thermodisinfected cancellous bone might improve compactness of the bone graft distal to the femoral stem and reduce the risk of subsidence (Cornu et al. 2003, 2004; Goldman and Sierra 2017; Kligman et al. 2003). A mixture of thermodisinfected bone chips of variable size between 3 and 10 mm should be preferred in the distal region below and near the tip of the stem. Native and thermodisinfected cancellous bone might be favorable in different regions of femoral impaction bone grafting (Frei et al. 2005b; Ohashi et al. 2009). The low applied torque has to be taken into account regarding implications for clinical application since impaction behavior might be different with higher loads. Multiple factors influencing the reproducibility of bone impaction have to be considered.

## Conclusion

Impaction of thermodisinfected cancellous bone showed a significant increased shear force resistance compared with native bone. Addition of antibiotic carrier material (Herafill®G) of small and varying size to both groups reduced that difference which was further influenced supplementing large granules. Thermodisinfected cancellous bone should be an alternative to native cancellous bone for impaction bone grafting since increased impaction seems to compensate mechanic alteration related to thermodisinfection. The relative micromovement within the native and thermodisinfected impacted bone graft appeared uniformly reduced in the distal region more obvious for thermodisinfected bone indicating different impaction behavior. Thermodisinfected cancellous bone chips size 3 mm to 10 mm could increase compactness of femoral impaction bone grafting distal and might therefore improve primary mechanical stability.

Small granules added to thermodisinfected bone revealed a marginal reduction of shear force resistance since a significant difference to native bone was still retained. Adding large granules to native cancellous bone improved shear force resistance significantly and no significant difference to the mixture of thermodisinfected bone with small granules was shown. The use of small antibiotic carrier particles with a range between 2 and 5 mm should be recommended for thermodisinfected impacted bone in particular since larger particles measuring 5 mm to 6 mm appeared preferrable to supplement native cancellous bone achieving comparable shear force resistance.
